# BLIMP1 Is Required for Postnatal Epidermal Homeostasis but Does Not Define a Sebaceous Gland Progenitor under Steady-State Conditions

**DOI:** 10.1016/j.stemcr.2014.08.007

**Published:** 2014-09-18

**Authors:** Kai Kretzschmar, Denny L. Cottle, Giacomo Donati, Ming-Feng Chiang, Sven R. Quist, Harald P. Gollnick, Ken Natsuga, Kuo-I Lin, Fiona M. Watt

**Affiliations:** 1 Centre for Stem Cells and Regenerative Medicine, King’s College London, Guy’s Hospital, 28^th^ Floor Tower Wing, Great Maze Pond, London SE1 9RT, UK; 2 Wellcome Trust-Medical Research Council Stem Cell Institute, University of Cambridge, Tennis Court Road, Cambridge CB2 1QR, UK; 3 Department of Genetics, University of Cambridge, Downing Street, Cambridge CB2 3EH, UK; 4 Department of Biochemistry and Molecular Biology, Monash University, Building 76, Wellington Road, Clayton, VIC 3800, Australia; 5 Epithelial Cell Biology Laboratory, Cancer Research UK Cambridge Research Institute, Li Ka Shing Centre, Robinson Way, Cambridge CB2 0RE, UK; 6 Graduate Institute of Life Sciences, National Defense Medical Centre, Taipei 114, Taiwan, ROC; 7 Genomics Research Center, Academia Sinica, Taipei 115, Taiwan, ROC; 8 Clinic of Dermatology and Venereology, Otto-von-Guericke University, Magdeburg, Leipziger Strasse 44, 39120 Magdeburg, Germany; 9 Department of Dermatology, Hokkaido University Graduate School of Medicine, North 15 West 7, Sapporo 060-8638, Japan

## Abstract

B-lymphocyte-induced nuclear maturation protein 1 (BLIMP1) was previously reported to define a sebaceous gland (SG) progenitor population in the epidermis. However, the recent identification of multiple stem cell populations in the hair follicle junctional zone has led us to re-evaluate its function. We show, in agreement with previous studies, that BLIMP1 is expressed by postmitotic, terminally differentiated epidermal cells within the SG, interfollicular epidermis, and hair follicle. Epidermal overexpression of *c-Myc* results in loss of BLIMP1^+^ cells, an effect modulated by androgen signaling. Epidermal-specific deletion of *Blimp1* causes multiple differentiation defects in the epidermis in addition to SG enlargement. In culture, BLIMP1^+^ sebocytes have no greater clonogenic potential than BLIMP1^−^ sebocytes. Finally, lineage-tracing experiments reveal that, under steady-state conditions, BLIMP1-expressing cells do not divide. Thus, rather than defining a sebocyte progenitor population, BLIMP1 functions in terminally differentiated cells to maintain homeostasis in multiple epidermal compartments.

## Highlights

• BLIMP1 is an epidermal terminal differentiation marker
• Loss of *Blimp1* perturbs differentiation in all epidermal lineages
• BLIMP1^+^ cells do not show enhanced clonal growth in culture
• BLIMP1^+^ cells are not sebocyte progenitors

## Teaser

In this article, Watt and colleagues re-examine the role of the transcription factor BLIMP1 in mammalian epidermis. They present evidence that, rather than defining a sebocyte progenitor population, BLIMP1-expressing cells do not divide under steady-state conditions. Rather, BLIMP1 functions in terminally differentiated cells to maintain homeostasis in multiple epidermal compartments.

## Introduction

Mammalian epidermis is maintained by stem cells that self-renew and give rise to the differentiated cells of the interfollicular epidermis (IFE), sebaceous glands (SGs), hair follicles (HFs), and sweat glands (Kretzschmar and Watt, 2014). Several different epidermal stem cell pools have been identified, including multiple HF stem cell populations. Under steady-state conditions, stem cells in different regions of the epidermis only give rise to the differentiated cells appropriate for their location, but when the epidermis is damaged or genetically modified, individual stem cells exhibit a broader ability to differentiate into all epidermal lineages (Watt and Jensen, 2009).

Within the epidermis, the differentiated cells of the SG produce sebum that lubricates and waterproofs the skin surface (Zouboulis et al., 2008). The specialized SGs of the eyelid (meibomian gland) and male genitals (preputial gland) contribute to the composition of the tears and secrete pheromones, respectively (House et al., 2010). SG dysfunction results in benign conditions, such as acne and sebaceous cysts, and also in a range of different tumor types. In vivo lineage tracing by retroviral transduction has established that the SG can be maintained by a population of long-lived progenitors (putative stem cells) that are distinct from the stem cells of the HF (Ghazizadeh and Taichman, 2001). The only specific marker of sebocyte progenitors to be described is B-lymphocyte-induced nuclear maturation protein 1 (BLIMP1) (also known as PR domain zinc finger protein 1 [PRDM1]; Horsley et al., 2006).

First identified as a gene upregulated during, and capable of promoting, terminal differentiation of B lymphocytes (Turner et al., 1994), BLIMP1 was subsequently characterized in many other tissues, mainly as a transcriptional regulator of terminal differentiation (Bikoff et al., 2009; John and Garrett-Sinha, 2009). During embryonic skin development, BLIMP1 expression was identified in the upper differentiated layers of the IFE and in differentiated cells of the HF inner root sheath (Chang et al., 2002). It was subsequently reported that BLIMP1 is also expressed in terminally differentiated cells of the IFE and SG of postnatal human and mouse skin and is upregulated in differentiating sebocytes in culture (Cottle et al., 2013; Lo Celso et al., 2008; Magnúsdóttir et al., 2007; Sellheyer and Krahl, 2010). In addition, by employing a range of experimental strategies, including immunohistochemistry, genetic lineage tracing, and cell culture, Fuchs and coworkers described BLIMP1 to be a marker of sebocyte progenitors (Horsley et al., 2006). In view of the importance of the SG in skin biology and new reports that cells expressing leucine-rich repeats and immunoglobulin-like domain protein 1 (LRIG1) or leucine-rich repeat-containing G-protein-coupled receptor 6 (LGR6) are SG progenitors (Jensen et al., 2009; Page et al., 2013; Snippert et al., 2010), we have revisited the function of epidermal BLIMP1.

## Results

### BLIMP1 Is Expressed by Terminally Differentiated Cells of the IFE, HF, and SG

**Figure 1:**
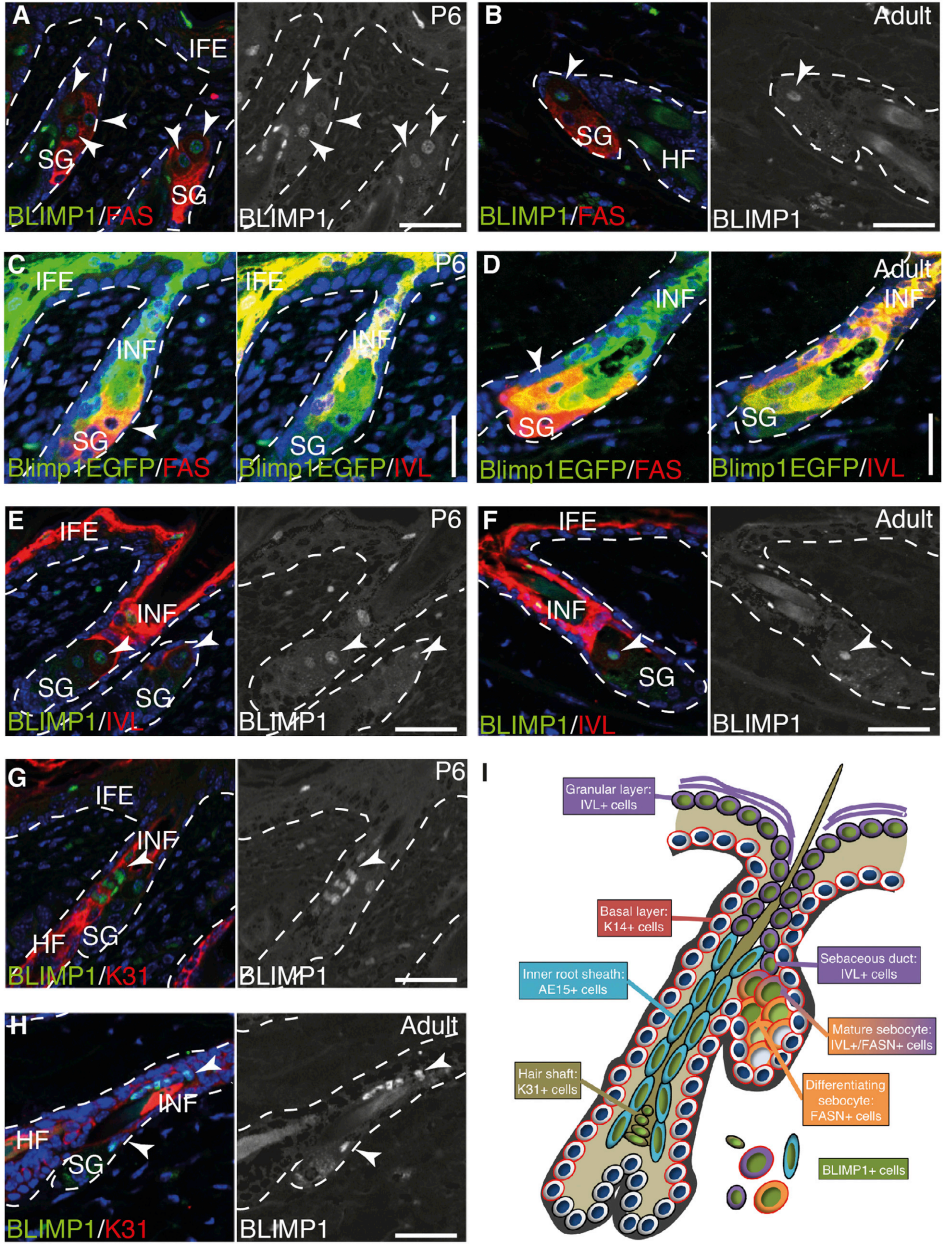
BLIMP1 Is Expressed by Terminally Differentiated Epidermal Cells in IFE, SG, and HF (A–H) Paraffin sections of murine wild-type or Blimp1EGFP (as indicated) back skin collected at postnatal day (P)6 or P56 (adult) labeled with antibodies against BLIMP1 (green in A, B, and E–H), GFP (green in C and D), FAS (red in A, B, and left panels of C and D), and involucrin (IVL) (red in E, F, and right panels of C and D) and counterstained with DAPI (blue). Black and white panels show BLIMP1 channel separately. Arrowheads, BLIMP1^+^ cells; dashed lines, epidermal-dermal boundary. (I) Summary of epidermal BLIMP1 expression. HF, hair follicle; IFE, interfollicular epidermis; INF, infundibulum; SG, sebaceous gland. Scale bars represent 50 μm. See also Figure S1.

We stained back skin sections of wild-type mice and transgenic mice expressing enhanced GFP (EGFP) under the control of the *Blimp1* promoter (Blimp1EGFP) (Ohinata et al., 2005) from different postnatal stages for endogenous BLIMP1 (Figure 1 and Figure S1 available online). In agreement with previous publications, BLIMP1 was localized to cell nuclei (Horsley et al., 2006; Magnúsdóttir et al., 2007; Robertson et al., 2007). Specific cells within all epidermal compartments (IFE, HF, and SG) expressed BLIMP1 (Figures S1A–S1D). As reported previously (Coulombe and Bernot, 2004; Coulombe et al., 1989), the entire SG expressed keratin 14 (K14) (Figure S1D). Cells double positive for BLIMP1 or Blimp1EGFP and the marker of differentiated sebocytes, fatty acid synthase (FAS), were found in the upper SG (Figures 1A–1D). BLIMP1 expression by FAS^+^ sebocytes was evident as soon as the SG began to develop at postnatal day (P)2 (Figures S1A–S1D). BLIMP1^+^ involucrin (IVL)^+^ cells as well as Blimp1EGFP^+^ IVL^+^ (Figures 1C–1F) were found in the sebaceous duct, which sits like a cap atop the SG and is an elongation of the HF infundibulum/junctional zone (Cottle et al., 2013). In the IFE, BLIMP1^+^ cells were absent from the K14^+^ basal layer and were found in the terminally differentiated, IVL^+^ cells of the granular layers (Figures 1E, 1F, and S1A–S1D). We confirmed the existence of a population of BLIMP1^+^ cells in the upper HF adjacent to the SG. BLIMP1^+^ cells in that region coexpressed IVL and the HF shaft differentiation marker K31, indicating that they were undergoing terminal differentiation (Figures 1G and 1H). The location of BLIMP1-expressing cells in the epidermis is summarized in Figure 1I.

We also stained sections of murine meibomian glands and preputial glands, which are specialized SGs (Figures S1E–S1H) (House et al., 2010). BLIMP1^+^ cells were found in the center of the meibomian glands, where the most highly differentiated sebocytes reside; they expressed FAS (Figures S1E and S1F), as reported previously (Cottle et al., 2013). Cells expressing BLIMP1 in preputial glands were also FAS^+^, confirming that they are indeed lipid-producing, differentiated sebocytes (Figures S1G and S1H). BLIMP1^+^ cells were also detected in the supporting HF ductal structures, coexpressing IVL (Figure S1H). Consistent with the observations in mouse skin, in human skin BLIMP1 was expressed in terminally differentiated, IVL^+^ epidermal cells in IFE, SG, and HF (Figures S1I–S1K). In human sebaceous tumors, BLIMP1 was expressed by the most differentiated (IVL^+^ or weak FAS^+^) cells in the center of the neoplasm rather than in the periphery, where the most proliferative cells reside (Figures S1L–S1P). BLIMP1 is expressed in papillary dermal fibroblasts during normal skin development (Driskell et al., 2013; Lesko et al., 2013) and was also expressed in tumor stromal cells (Figures S1L–S1P).

### Modulation of BLIMP1 Expression by MYC and Androgens

**Figure 2:**
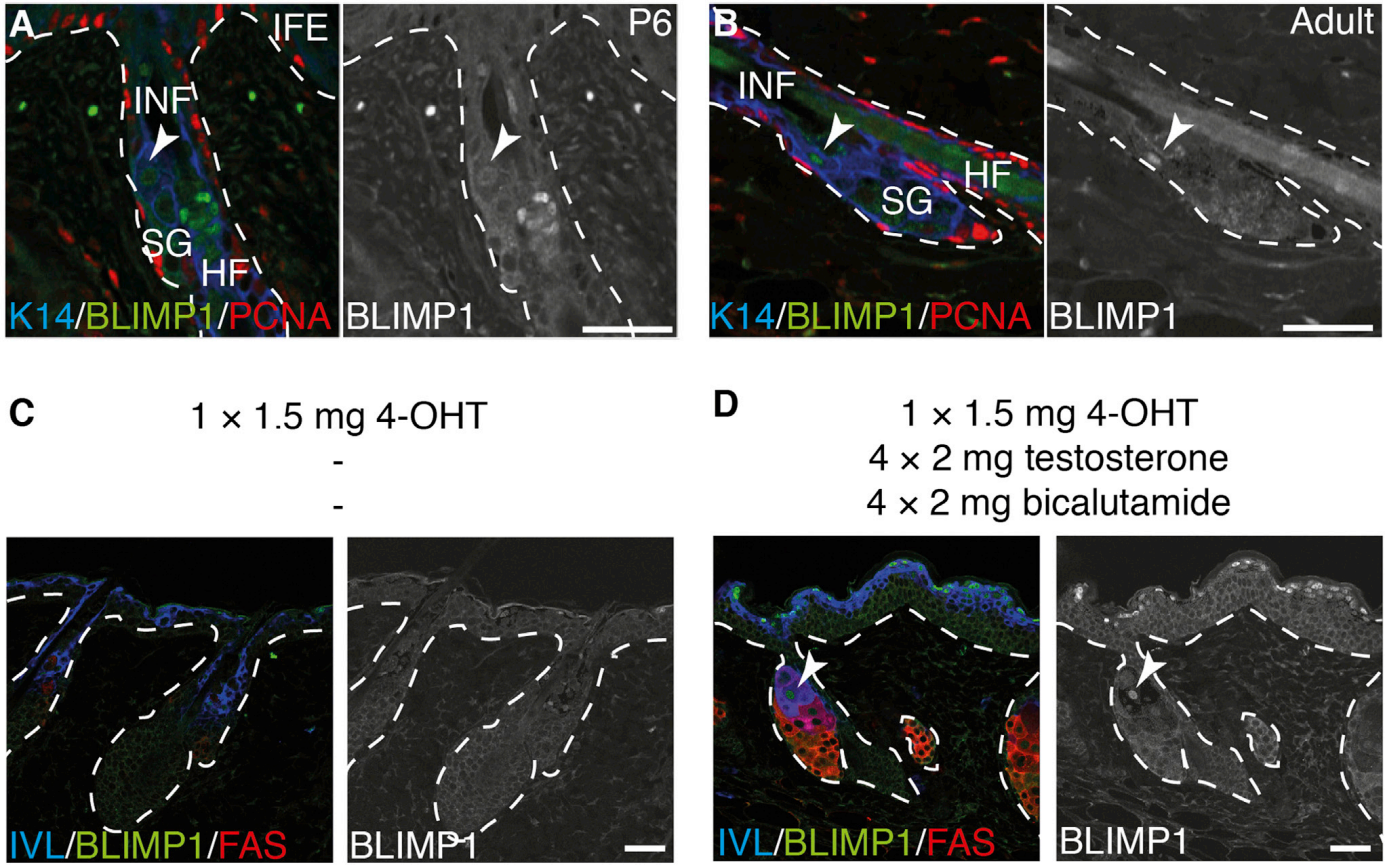
BLIMP1 Expression Is Regulated by MYC (A and B) Paraffin sections of murine wild-type back skin collected at P6 or P56 (adult) labeled with antibodies against BLIMP1 (green), PCNA (red), and K14 (blue). (C and D) Paraffin sections of adult K14c-MycER^t^ transgenic mice treated with 4-hydroxytamoxifen (4-OHT) alone (C) or in combination with testosterone and bicalutamide and labeled with antibodies against BLIMP1 (green), FAS (red), and IVL (blue). Black and white panels show BLIMP1 channel separately. Arrowheads, BLIMP1^+^ cells; dashed lines, epidermal-dermal boundary. Scale bars represent 50 μm (A and B) and 100 μm (C and D).

BLIMP1 has previously been shown to bind and negatively regulate the *c-Myc* promoter (Horsley et al., 2006), and epidermal *c-Myc* overexpression, like *Blimp1* deletion, can lead to SG enlargement (Horsley et al., 2006; Berta et al., 2010; Cottle et al., 2013). To determine the effect of *c-Myc* overexpression on *Blimp1* expression in the epidermis, we first confirmed that, in wild-type epidermis, BLIMP1^+^ cells did not express proliferating cell nuclear antigen (PCNA), a marker of proliferation (Figures 2A and 2B). When MYC was activated by high-dose 4-hydroxy-tamoxifen (4-OHT) treatment in K14c-MycER^t^ transgenic mice (Berta et al., 2010; Cottle et al., 2013), proliferation in the SG was stimulated and there was a reduction in BLIMP1^+^ SG cells (Figure 2C). In contrast, when MYC-induced SG proliferation was inhibited by the androgen testosterone and sebocyte terminal differentiation was stimulated by the antiandrogen bicalutamide (Cottle et al., 2013), there was an increase in the number of BLIMP1^+^ differentiated sebocytes (FAS^+^) and BLIMP1^+^ cells in the upper layers of the IFE (Figure 2D). These observations indicate that accumulation of BLIMP1^+^ cells in the SG is correlated with terminal differentiation rather than proliferation of SG progenitors.

### Epidermal Loss of *Blimp1* Causes Multiple Epidermal Deficiencies, Including Sebaceous Gland Enlargement

**Figure 3:**
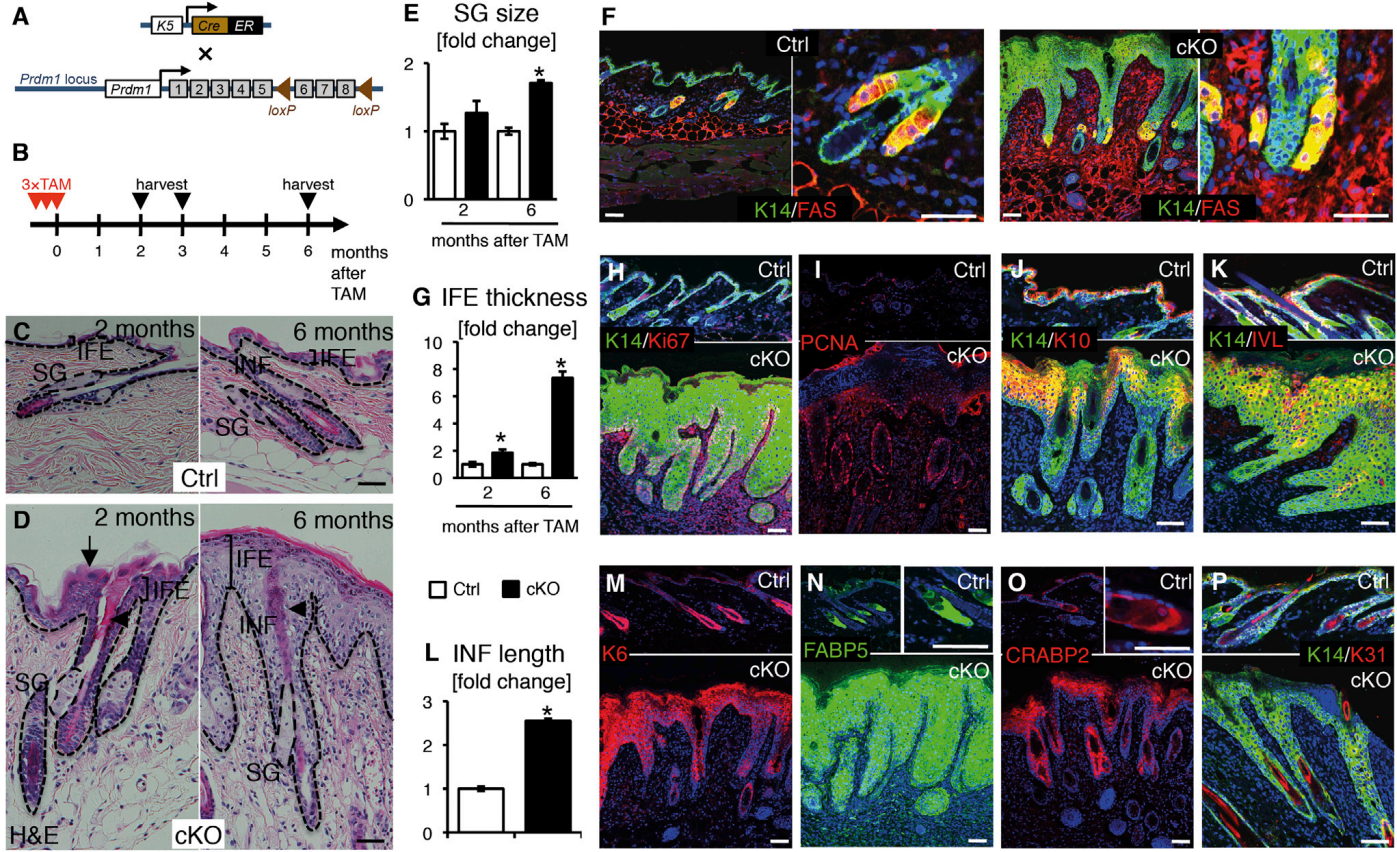
Epidermal-Specific Loss of *Blimp1* Causes Multiple Differentiation Defects (A and B) Schematics of genetic elements (A) and experimental setup (B). (C and D) Paraffin sections of neck skin collected from *Blimp1* conditional knockout (cKO) mice and control littermates 2 and 6 months after tamoxifen injection. Dashed lines indicate epidermal-dermal boundary and delineate area of SG quantified. Square brackets show IFE thickness quantified. Arrow indicates outermost cornified layers. Arrowheads indicate hyperkeratosis of HF infundibulum. (E) Quantitation of SG size in Blimp1 cKO epidermis normalized to control. Quantitation was performed on a minimum of five H&E-stained sections per mouse from three cKO and three control mice. (F) Paraffin sections stained with antibodies against FAS (red) and K14 (green) and counterstained with DAPI (blue). (G) Quantitation of IFE thickness in Blimp1 cKO epidermis normalized to control. Quantitation was performed on a minimum of five H&E-stained sections per mouse from three cKO and three control mice. (H–K) Paraffin sections stained with antibodies against Ki67 (red in H), PCNA (red in I), K10 (red in J), or IVL (red in K) and K14 (green in H, J, and K) and counterstained with DAPI (blue). (L) Quantitation of infundibulum length in Blimp1 cKO epidermis normalized to control. Quantitation was performed on a minimum of five H&E-stained sections per mouse from three cKO and three control mice. (M–P) Paraffin sections stained with antibodies against K6 (red in M), FABP5 (green in N), CRABP2 (red in O), or K31 (red in P) and counterstained with DAPI (blue). cKO, conditional knockout; TAM, tamoxifen. Error bars represent the SEM. Asterisks indicate significant differences between control and cKO (unpaired two-tailed Student’s t test; p values: ^∗^p < 0.05, ^∗∗^p < 0.005, ^∗∗∗^p < 0.001). Scale bars represent 100 μm, except those in the left-hand panels for Ctrl and cKO in (F), which represent 200 μm. See also Figure S2.

There are conflicting reports about the consequences of epidermal loss of *Blimp1* (*Blimp1* conditional knockout [cKO]). In one report, there were no obvious defects in the IFE or HF and specific SG abnormalities were found, including SG enlargement and hyperplasia in the skin, the meibomian glands, and the preputial glands (Horsley et al., 2006). In contrast, Magnúsdóttir et al. (2007) found that epidermal-specific deletion of *Blimp1* caused not only SG enlargement but also IFE hyperplasia, abnormal expansion of the granular layer, and a hyperkeratotic HF infundibulum. The IFE hyperplasia was absent in *Blimp1*-deficient epidermis in mice older than 15 days, and only SG enlargement and hyperkeratinization of the HF infundibulum persisted into adulthood (Magnúsdóttir et al., 2007). Both studies used the same K14Cre mouse strain; however, Magnúsdóttir et al. (2007) used a floxed *Blimp1* strain that deletes exons 6–8 upon recombination (*Prdm1*^tm1Clme^; Figure 3A) (Shapiro-Shelef et al., 2003) whereas Horsley et al. (2006) used an exon 5 floxed *Blimp1* strain (*Prdm1*^tm2Masu^; Ohinata et al., 2005).

In order to delete *Blimp1* selectively in adult, rather than developing, epidermis and therefore evaluate whether a BLIMP1^+^ progenitor population does indeed govern cellular input to the SG, we crossed *Prdm1*^tm1Clme^ mice with K5CreER^t^ transgenic mice (Liang et al., 2009) and induced Cre-mediated deletion of *Blimp1* in adult mice by injecting tamoxifen (Figures 3A and 3B) (Chiang et al., 2013). Deletion of *Blimp1* in the entire epidermis was confirmed as reported previously (Figure S2) (Chiang et al., 2013). Hematoxylin and eosin (H&E) staining of neck skin sections collected 2, 3, and 6 months after tamoxifen injection revealed clear differences between control and *Blimp1* cKO epidermis, with epidermal loss of *Blimp1* resulting in SG enlargement, IFE thickening, accumulation of cornified layers, and hyperkeratosis of the HF infundibulum (Figures 3C and 3D). Although our analysis was restricted to neck skin, other studies have shown no evidence for differences between neck and back skin (Magnúsdóttir et al., 2007; Chiang et al., 2013). The phenotype resembled that described by Magnúsdóttir et al. (2007) in neonatal *Blimp1*^−/−^ epidermis rather than a SG-specific phenotype (Horsley et al., 2006).

Quantification of H&E-stained sections showed that *Blimp1* deletion resulted in a gradual increase in SG size over time (Figure 3E). Sections of *Blimp1* cKO tissue stained for FAS showed no obvious changes in expression within sebocytes (Figure 3F). *Blimp1*-deficient IFE was significantly thickened (Figure 3G) and showed a dramatic increase in Ki67^+^/PCNA^+^ basal layer cells in the IFE and HF infundibulum (Figures 3H and 3I). K14, a marker that is usually restricted to the basal layer in the IFE, expanded into the suprabasal layers (Figure 3H), as reported in neonatal cKO skin (Magnúsdóttir et al., 2007). K10 and IVL, markers of IFE differentiation, were expressed in the suprabasal layers, but the strong overlap of those markers with K14 indicates perturbed IFE differentiation (Figures 3J and 3K). We also found that the infundibulum of adult *Blimp1* cKO skin displayed increased proliferation and was hyperkeratotic, thickened, and elongated (Figures 3D and 3L).

Markers typical of epidermal hyperproliferation, namely K6, the retinoic acid (RA)-signaling molecule fatty-acid-binding protein, FABP5, and cellular RA-binding protein, CRABP2 (the latter two being also sebocyte markers in normal skin; Collins and Watt, 2008), were also dramatically increased in *Blimp1* cKO epidermis (Figures 3M–3O). These changes are consistent with a defective epidermal barrier (Magnúsdóttir et al., 2007) and would explain the massive inflammatory infiltrate in the dermis (Figure 3D) (Chiang et al., 2013). Staining for the hair shaft marker K31 did not reveal any abnormalities in the lower, cycling portion of the HF (Figure 3P) (Horsley et al., 2006; Magnúsdóttir et al., 2007).

We conclude that epidermal loss of *Blimp1* expression in adult epidermis causes multiple epidermal defects, including SG enlargement, hyperplasia, and perturbed differentiation of the IFE and HF infundibulum.

### All Sebocytes Retain Proliferative Potential in Culture

**Figure 4:**
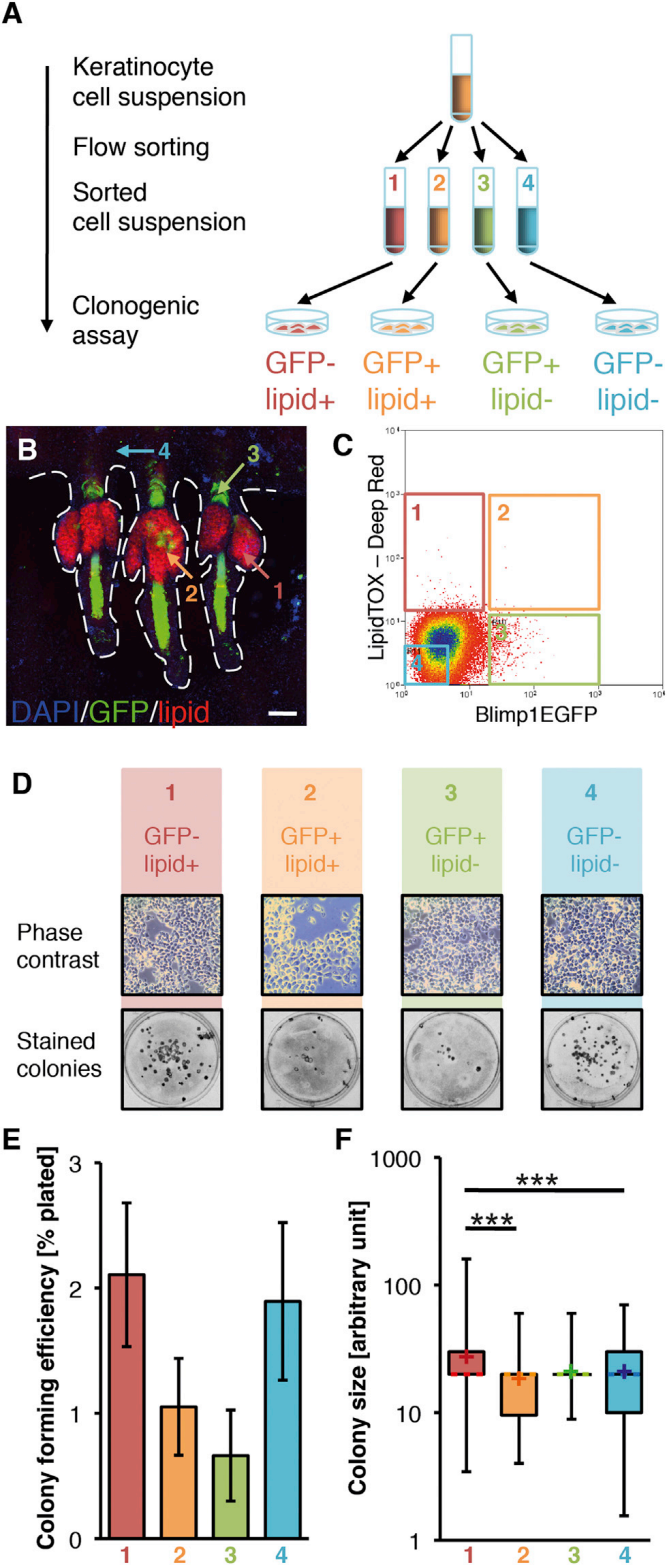
Clonogenic Potential of BLIMP1^+^ Sebocytes (A) Schematic of experimental setup. (B) Epidermal tail whole mount of Blimp1EGFP mouse stained with GFP antibody and LipidTOX to visualize lipid-producing cells; counterstained with DAPI (blue). Dashed line outlines HF and SG. Numbers correspond to sorted populations in (C). (C) Flow cytometry plot showing sorted cell populations. (D) Phase contrast images (top) and stained dishes (bottom) of mouse epidermal cells after 14 days in culture (after removal of feeder layer). (E and F) Colony-forming efficiency (E) and average colony size (F; whisker plot) of sorted populations. Error bars in (E) show SEM. In (F), vertical lines show 25% confidence intervals; means are indicated by crosses and medians by dashed lines. (E) and (F) data are biological replicates (n = 3 mice). Asterisks indicate significance of differences between cell populations (unpaired two-tailed Student’s t test; p values: ^∗∗∗^p < 0.001). The scale bar represents 100 μm.

BLIMP1^+^ epidermal cells have the ability to proliferate at clonal density and give rise to lipid-filled sebocytes (Horsley et al., 2006). In order to compare the proliferative ability of BLIMP1^+^ sebocytes and other epidermal cells, we first incubated adult back skin keratinocytes from Blimp1EGFP mice with LipidTOX dye (Life Technologies) to label lipid-producing sebocytes. We then flow sorted four populations of keratinocytes: (1) EGFP^−^lipid^+^ sebocytes; (2) EGFP^+^lipid^+^ sebocytes; (3) EGFP^+^lipid^−^ cells (comprising BLIMP1^+^ cells of IFE, HF, and sebaceous duct); and (4) EGFP^−^lipid^−^ cells (comprising primarily undifferentiated cells and BLIMP1^−^ differentiated cells; Figures 4A–4C). Sorted cells were cultured on a feeder layer, and colony-forming efficiency (CFE) was analyzed 2 weeks later.

Lipid^+^ sebocytes, whereas not proliferative in vivo, were clonogenic at low frequency, confirming previous studies (Laurent et al., 1992). EGFP^+^ sebocytes did not show a significantly increased CFE compared to EGFP^−^ sebocytes (Figures 4D–4F). EGFP^+^ lipid^−^ cells displayed a reduced CFE compared to EGFP^−^lipid^−^ cells, although this was not statistically significantly (Figure 4E). Analysis of colony sizes revealed EGFP^−^lipid^+^ sebocytes formed the largest colonies in comparison to the other three cell populations (Figure 4F). We conclude that BLIMP1^+^ cells, whether or not they are sebocytes, have a lower clonogenic capacity than BLIMP1^−^ cells, consistent with their differentiated status.

### BLIMP1^+^ Cells Do Not Give Rise to Proliferative and Differentiating Sebocytes In Vivo

**Figure 5:**
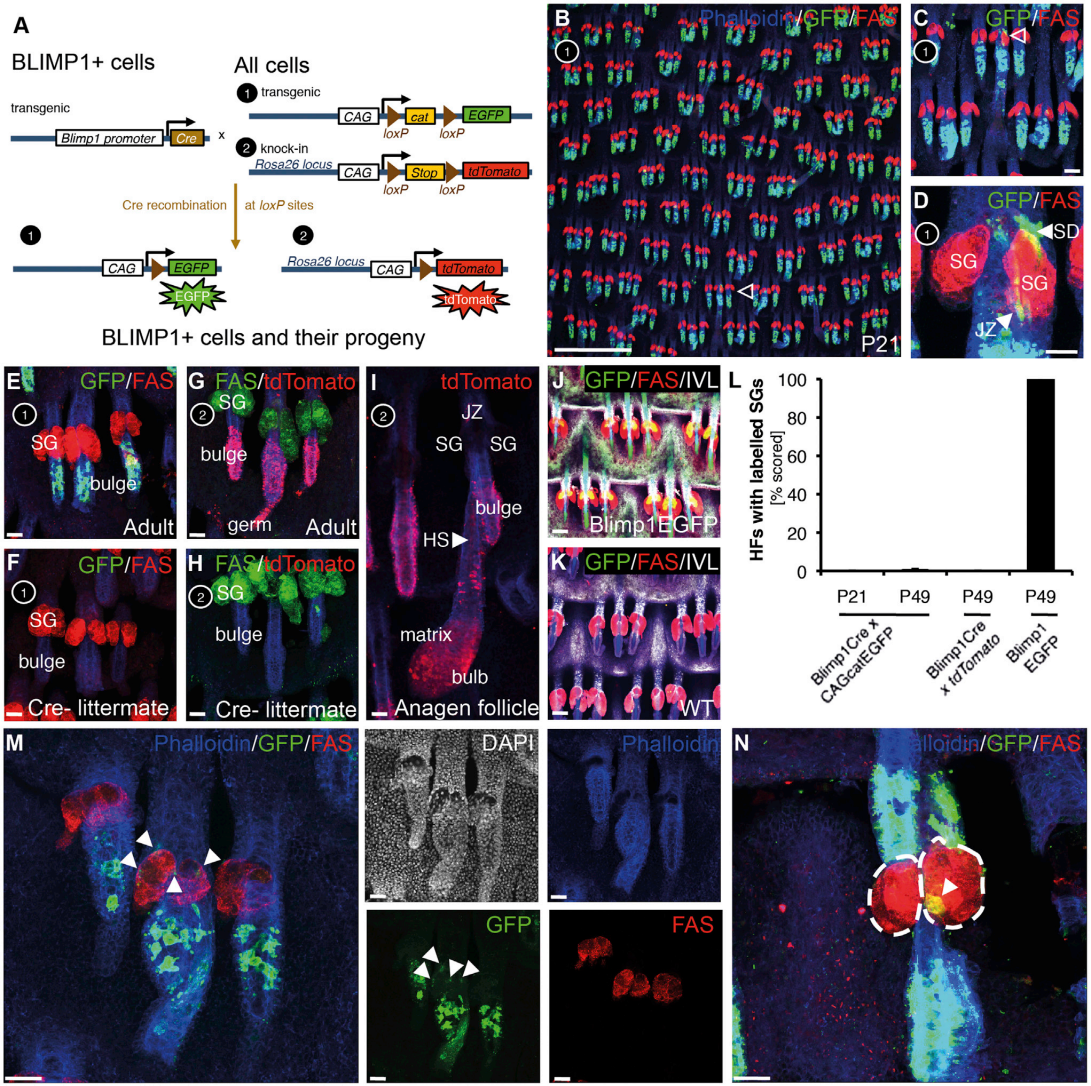
Lineage Tracing the Progeny of BLIMP1^+^ Cells (A) Schematic of lineage-tracing experiments. Numbers correspond to labeling strategies in (B)–(I). (B–F) Epidermal tail whole mounts collected from Blimp1Cre × CAGcatEGFP mice at P21 (B–D) and P49 (E) and control mice (F) stained with antibodies against GFP (green) and FAS (red) and counterstained with phalloidin (blue). (G–I) Epidermal tail whole mounts collected from Blimp1Cre × *Rosa26tdTomato* mice at P49 (G and I) and control mice (H) stained with antibodies against tdTomato (red) and FAS (green) and counterstained with phalloidin. (J and K) Epidermal tail whole mounts collected from Blimp1GFP mice at P49 (J) and control mice (K) stained with antibodies against GFP (green), FAS (red), and IVL (white). (L) Quantitation of percent HFs with fluorescent-reporter-labeled SGs. Quantification was performed on stained 0.5 × 0.5 cm epidermal tail whole mounts collected from three mice per strain and time point. (M and N) Epidermal tail whole mounts collected from Blimp1Cre × CAGcatEGFP mice at P49 and stained with antibodies against GFP (green) and FAS (red), counterstained with phalloidin (blue) or DAPI (white in M). SD, sebaceous duct; JZ, junctional zone; HS, hair shaft; arrowheads in (B) and (C), SG; arrowheads in (M) and (N), single GFP^+^ cells. Scale bars represent 100 μm, except the scale bar in (B), which represents 200 μm. See also Figure S3.

In order to understand whether a subset of BLIMP1^+^ cells gives rise to differentiated sebocytes, we performed genetic lineage-tracing experiments (Kretzschmar and Watt, 2012). We crossed Blimp1Cre transgenic mice (Ohinata et al., 2005; Horsley et al., 2006) with chicken β-actin promoter and cytomegalovirus (CMV) enhancer-chloramphenicol acetyltransferase-EGFP (CAGcatEGFP) transgenic (Kawamoto et al., 2000) or *Rosa26tdTomato* (Ai9 line) gene trap (Madisen et al., 2010) reporter mice (Figure 5A). By constitutively expressing Cre recombinase under the control of the *Blimp1* promoter, the floxed STOP cassette of the reporter construct is removed and the fluorescent reporter (EGFP or tdTomato) expressed in BLIMP1^+^ cells and their descendants (Figure 5A). If these *Blimp1*-expressing cells are indeed sebocyte progenitors, labeled progeny should be found throughout the SG in cells at all stages of differentiation.

We collected tissue from Blimp1Cre × CAGcatEGFP and Blimp1Cre *× Rosa26tdTomato* mice at weaning age (P21) and adult (P56) and stained tail epidermal whole mounts (Braun et al., 2003) with antibodies against the respective reporter and the sebocyte marker, FAS (Cottle et al., 2013). Neither EGFP nor tdTomato was significantly detected in FAS^+^ sebocytes (Figures 5B–5I and 5L). Clones expressing either reporter were found in differentiated cells of the inner bulge, as well as in the matrix of anagen HFs (Figures 5B–5I). Figure 5D shows that rare EGFP^+^ clones, containing a small number of cells, were present in the HF junctional zone and sebaceous duct, but not in basal or FAS^+^ sebocytes. These lineage-tracing results largely mirror the expression of endogenous BLIMP1 and Blimp1EGFP in all epidermal lineages (Figures 1, 5J, and 5K). Labeled progeny adjacent to the SG were mainly found in the inner layers of the HF junctional zone, and only one EGFP^+^ traced cell in the epidermis of all mice examined expressed FAS (Figures 5M and 5N). Similar data were obtained in horizontal whole mounts of back epidermis, which also show EGFP^+^ lineage traced clones in the differentiated layer of IFE (overlapping with IVL) and HF (Figure S3). Dermal labeling is also observed in line with recent studies on the role of BLIMP1 in dermal fibroblast subpopulations (Driskell et al., 2013; Lesko et al., 2013).

In Blimp1EGFP mice, EGFP is expressed by cells that are expressing endogenous BLIMP1, regardless of whether they are derived from BLIMP1^+^ cells (Lesko et al., 2013). In contrast, in Blimp1Cre × *Rosa26tdTomato* mice, tdTomato expression (and likewise Blimp1Cre × CAGcatEGFP mice EGFP expression) is restricted to cells that are BLIMP1^+^ and their progeny. The cells that give rise to the BLIMP1^+^ cells of the IFE and SG were BLIMP1^−^ (e.g., IFE basal layer) and so were labeled with EGFP and not tdTomato. Of note, cells in the upper layers of the IFE cannot be visualized in tail epidermal whole mounts, because antibodies cannot penetrate deeper than the first suprabasal layer of the fixed tissue. However, in horizontal whole mount sections, EGFP and tdTomato labeling in the IFE and SG was clearly visible (Figure S3; data not shown). The lack of tdTomato labeling in terminally differentiated sebocytes may reflect the short half-life of the cells. The lack of EGFP- or tdTomato-labeled basal cells and early sebocytes supports the conclusion that BLIMP1 does not define a sebocyte progenitor population.

### LGR6^+^ and LRIG1^+^ Stem Cells Contribute to Maintenance of the Sebaceous Gland

**Figure 6:**
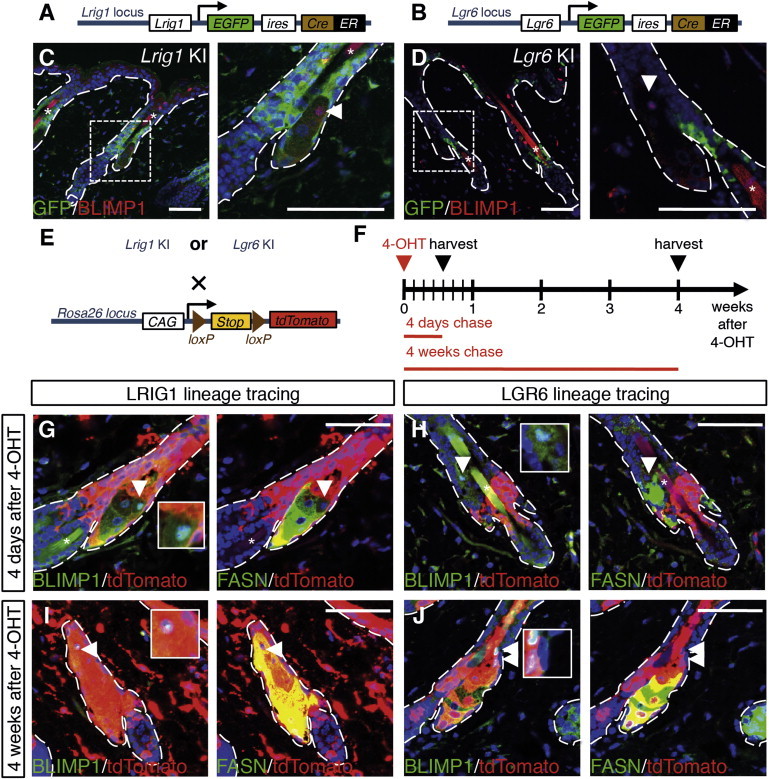
Lineage Tracing the Progeny of LRIG1^+^ and LGR6^+^ Stem Cells (A and B) Genetic elements of the *Lrig1* knockin (KI) and *Lgr6* KI. (C and D) Paraffin sections of adult *Lrig1* KI (C) and *Lgr6* KI (D) back skin stained with antibodies against GFP (green) and BLIMP1 (red), counterstained with DAPI (blue). Boxed area is shown at higher magnification. (E and F) Schematic of experimental breeding and procedures. (G–J) Paraffin sections of *Lrig1* KI × Rosa26tdTomato (G and I) and *Lgr6* KI × Rosa26tdTomato (H and J) collected 4 days (G and H) and 4 weeks (I and J) after one dose of 4-OHT and stained for BLIMP1 (green, left panels) or FAS (green, right panels) and tdTomato (red). Arrowheads indicate BLIMP1^+^ cells. Dashed lines indicate epidermal-dermal boundary. Scale bars represent 100 μm. See also Figure S4.

LRIG1 and LGR6 have previously been shown to be markers of epidermal stem cells in the HF junctional zone that produce progeny in the SG (Figures 6A and 6B) (Jensen et al., 2009; Page et al., 2013; Snippert et al., 2010). We stained tissue sections of adult *Lrig1EGFPiresCreER*^T2^ (Page et al., 2013) and *Lgr6EGFPiresCreER*^T2^ (Snippert et al., 2010) knockin (KI) mice with antibodies against EGFP (to label the respective stem cell pools) and endogenous BLIMP1. In agreement with the earlier findings, we found that BLIMP1 was not coexpressed with LRIG1 or LGR6 (Figures 6A–6D).

To confirm that our lineage-tracing strategy was capable of labeling the SG lineage and to rule out any possible effects of using the *CMV* and *CAG* promoter versus *Rosa26* promoters or different fluorescent reporters, we crossed our *Rosa26tdTomato* strain (Madisen et al., 2010) with *Lrig1* KI and *Lgr6* KI mice (Figure 6E). Offspring positive for either of the two *EGFPiresCreER*^T2^ cassettes and the tdTomato reporter were treated with 1.5 mg 4-OHT at 7–9 weeks of age, and back skin was examined 4 days and 4 weeks later (Figure 6F). This dose of 4-OHT achieved maximal labeling of the stem cell pools. Paraffin sections were stained for tdTomato, BLIMP1, and FAS.

Four days after 4-OHT application, tdTomato^+^ LRIG1 stem cell progeny were found in the periphery and lower SG, as well as in some sebaceous duct cells. However, BLIMP1^+^ sebocytes were negative for the reporter (Figure 6G). As *Lrig1* is also expressed in the dermis (Driskell et al., 2013; Gomez et al., 2013), tdTomato^+^ dermal LRIG1 progeny were also observed. TdTomato^+^ LGR6 stem cell progeny were also found in the lower SG, but again tdTomato labeling was absent from BLIMP1^+^ sebocytes (Driskell et al., 2013; Figure 6H). Four weeks after 4-OHT treatment, some BLIMP1^+^ sebocytes were also tdTomato^+^ (Figures 6I and 6J), establishing that, between 4 days and 4 weeks after labeling, progeny of LRIG1^+^ and LGR6^+^ stem cells underwent terminal differentiation into BLIMP1^+^ sebocytes. Of note, even with the high dose of 4-OHT applied, SGs of *Lgr6* KI × *Rosa26tdTomato* mice were frequently labeled in the absence of isthmus labeling (Figure S4), strongly suggesting the existence of sebocyte progenitor residing within the SG, as proposed previously (Page et al., 2013).

In conclusion, our results confirm that the SG lineage is indeed derived from LRIG1^+^ and LGR6^+^ stem cells (Jensen et al., 2009; Page et al., 2013; Snippert et al., 2010) and that BLIMP1^+^ SG cells are terminally differentiating sebocytes (Cottle et al., 2013).

## Discussion

Although the concept that BLIMP1 is a marker of sebocyte progenitor cells has become established in the literature (Beck and Blanpain, 2012; Blanpain and Fuchs, 2014; Niemann and Horsley, 2012; Solanas and Benitah, 2013; Zhang et al., 2011), our studies suggest that BLIMP1 is primarily a marker of terminal differentiation in SG, IFE, and HF and that BLIMP1^+^ cells do not divide in undamaged postnatal epidermis. Nevertheless, genetic ablation of BLIMP1 confirms its importance in epidermal homeostasis, and clonogenic assays demonstrate that BLIMP1^+^ sebocytes, whereas nonproliferative in vivo, can divide in vitro.

We showed that specific deletion of *Blimp1* caused defects in differentiation in multiple epidermal compartments, namely the IFE, SG, HF infundibulum, and junctional zone. Hyperplasia was not restricted to the SG and was more pronounced in the IFE and infundibulum. The aberrant IFE differentiation was suggestive of a barrier defect, which would explain the previously reported inflammatory skin phenotype (Chiang et al., 2013). The two previously published studies on epidermal deletion of *Blimp1* in early development (Horsley et al., 2006; Magnúsdóttir et al., 2007) used a K14Cre mouse described by Vasioukhin et al. (1999) to target the epidermal basal layer but used different conditional alleles of *Blimp1*: Horsley et al. (2006) used an exon 5 floxed *Blimp1* strain (*Prdm1*^tm2Masu^; Ohinata et al., 2005), whereas Magnúsdóttir et al. (2007) used a floxed *Blimp1* strain with loxP sites between exons 6–8 (*Prdm1*^tm1Clme^; Figure 3A) (Shapiro-Shelef et al., 2003). Both studies reported SG enlargement (Horsley et al., 2006; Magnúsdóttir et al., 2007), a phenotype that we also observed on epidermal deletion of *Blimp1* in adult mice. In addition, Magnúsdóttir et al. (2007), Chiang et al. (2013), and the present study found thickening of the IFE and HF infundibulum, regardless of whether *Blimp1* was deleted in the embryo or adult. Potential reasons for the divergent observations regarding whether or not *Blimp1* has a selective role in the SG include differences in the genetic background of the mice, animal husbandry (diet, health status, and pathogens), or the presence of truncated BLIMP1 protein that is undetectable with current antibodies (Bikoff et al., 2009). Differences in Cre expression over time could also contribute, as suggested for other transgenic Cre lines (Kang et al., 2014).

BLIMP1 is a transcriptional repressor of *c-Myc* (Lin et al., 1997), and forced activation of MYC resulted in downregulation of BLIMP1 and increased proliferation within the SG (Cottle et al., 2013). We have previously observed that androgen receptor signaling modulates epidermal responses to MYC activation (Cottle et al., 2013), and consistent with this, stimulation of sebocyte differentiation was accompanied by an increase in the number of BLIMP1^+^ cells. BLIMP1 repression of *c-Myc* may also contribute to the transition of IFE cells out of the granular layer (Honma et al., 2006). Consistent with BLIMP1 being associated with terminal differentiation, BLIMP1 expression was downregulated in human SG tumors. Another negative regulator of BLIMP1 is miR-125b, which is upregulated in stem cells and progenitors in the HF and SG (Zhang et al., 2011).

For lineage-tracing experiments, we crossed Blimp1Cre transgenic mice with two different *loxP-STOP-loxP* fluorescent reporter strains. We found no evidence that BLIMP1^+^ cells gave rise to differentiated sebocytes or indeed any labeled progeny. Instead, subpopulations of *Lgr6*- and *Lrig1*-expressing cells founded the sebocyte lineage, as reported previously (Jensen et al., 2009; Page et al., 2013; Snippert et al., 2010). Although we used the same Blimp1Cre line as in the earlier lineage-tracing experiments (Horsley et al., 2006; Ohinata et al., 2005), we utilized different reporter lines, transgenic CAGcatEGFP and gene-trap *Rosa26tdTomato* (used in our study) compared to gene-trap *Rosa26EYFP* (used in the study by Horsley et al., 2006), which have been shown to be more sensitive (Duffield and Humphreys, 2011; Kawamoto et al., 2000; Kretzschmar and Watt, 2012; Madisen et al., 2010). The fluorescent lineage tracers we used are expressed more strongly upon recombination, due to expression driven from the *CAG* promoter rather than the weaker *Rosa26* promoter used by Horsley et al. (2006; Kawamoto et al., 2000; Soriano, 1999; Srinivas et al., 2001). EGFP and tdTomato also have the spectral advantage over EYFP when distinguishing true epifluorescence from highly autofluorescent structures such as the lipid-rich SG and sebaceous duct.

**Figure 7:**
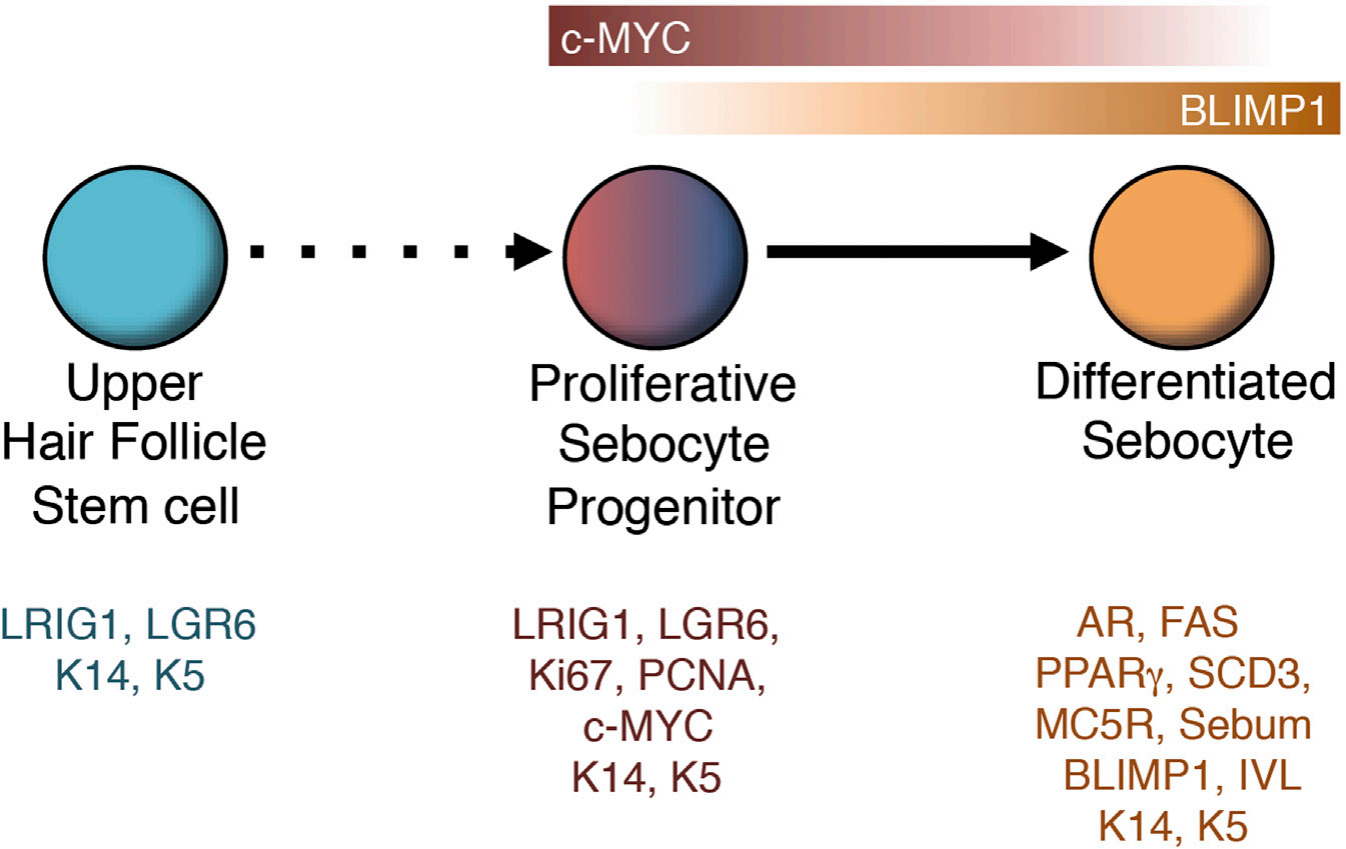
Model of Sebocyte Differentiation

Based on our findings and the recent literature, we propose the following model of sebocyte differentiation (Figure 7). First, subsets of LGR6- and LRIG1-expressing cells residing in the upper HF and periphery of the SG constitute the bona fide stem cells of the sebocyte lineage (Page et al., 2013; Snippert et al., 2010). This is in line with the observation that the SG can be maintained independently of the HF lineages (Ghazizadeh and Taichman, 2001). We do not rule out the existence of other SG stem cell compartments, and indeed, we believe this is likely, given the diversity of stem cells elsewhere in the epidermis (Kretzschmar and Watt, 2014). Second, we propose that MYC plays a role in proliferation of cells that are committed to undergo terminal differentiation. Third, upregulation of BLIMP1 promotes terminal differentiation by repressing *c-Myc* and inhibiting proliferation (Figure 7) (Berta et al., 2010; Cottle et al., 2013). Our revised model suggests that *Blimp1* cKO mice exhibit SG hyperplasia directly because of derepression of *c-Myc*, as reported previously (Horsley et al., 2006), and indirectly by causing a barrier defect in the IFE (Chiang et al., 2013), which activates stem cells within the HF infundibulum/junctional zone and SG periphery to proliferate (Page et al., 2013).

In conclusion, our findings indicate that the role of BLIMP1 in the epidermis is to maintain homeostasis in multiple compartments, including the SG, but that it exerts its effects in terminally differentiated cells rather than in sebocyte progenitors.

## Experimental Procedures

### Human Tissue

Human skin and tumors were collected and diagnosed by Dr. Harald P. Gollnick and Dr. Sven R. Quist from the Clinic of Dermatology and Venereology, Otto-von-Guericke University Magdeburg, Germany, and Dr. Ken Natsuga from the Department of Dermatology, Hokkaido University Graduate School of Medicine, Sapporo, Japan. Patient consent records and ethical review are retained by the respective institutions.

### Generation and Experimental Treatment of Mice

Mouse experiments were subject to Cancer Research UK, University of Cambridge, King’s College London, and Institutional Animal Care and Utilization Committee of Academia Sinica ethical review and performed in accordance with the UK Government Animals (Scientific Procedures) Act 1986.

Blimp1EGFP (Ohinata et al., 2005) transgenic and F1 (CBA × Bl6) wild-type mice were used for the initial characterization of BLIMP1 expression. To obtain mice with epidermal deletion of *Blimp1* (*Prdm1*), *Blimp1*^flox/flox^ (*Prdm1*^tm1Clme^; Shapiro-Shelef et al., 2003) and K5CreER^t^ mice (Liang et al., 2009) were crossed and tamoxifen treated as described previously (Chiang et al., 2013). For constitutive lineage-tracing experiments, Blimp1Cre mice (Ohinata et al., 2005) were either crossed with *Rosa26tdTomato* (Ai9 line) knockin (Madisen et al., 2010) or CAGcatEGFP transgenic (Kawamoto et al., 2000) reporter mice.

For 4-OHT (Sigma)-induced lineage-tracing, *Lgr6EGFPiresCreER*^T2^ (Snippert et al., 2010) and *Lrig1EGFPiresCreER*^T2^ (Page et al., 2013) knockin mice were bred to *Rosa26tdTomato* reporter mice. Their offspring were treated with one dose of 1.5 mg 4-OHT dissolved in 100 μl acetone applied to clipped back skin at 7–9 weeks of age, and tissue was collected 4 days and 4 weeks after 4-OHT application.

K14c-MycER^t^ (2184 C.1 line) transgenic mice (Arnold and Watt, 2001) were treated once with 1.5 mg 4-OHT and then subsequently with 2 mg testosterone and 2 mg bicalutamide or carrier only (acetone) for 4 days and analyzed 4 days after the first treatment (Cottle et al., 2013). At the start of every experiment, all the mice were 7–9 weeks old and therefore in the resting phase (telogen) of the hair cycle (Stenn and Paus, 2001). Wild-type littermates and acetone-only-treated transgenic mice were used as controls. At least three mice were treated per condition.

### Mouse Keratinocyte Isolation, Flow Cytometry, and Clonogenic Assays

Keratinocytes were isolated from telogen back skin of adult Blimp1EGFP mice using trypsin (Life Technologies), as previously described (Jensen et al., 2010). Isolated keratinocytes were incubated with LipidTOX dye (Life Technologies) diluted 1:500 in PBS for 20 min. Flow sorting was carried out using a MoFlo high-speed sorter (Dako Cytomation) or a FACSAria II cell sorter (BD Biosciences). Sorted keratinocytes were plated onto a J2 3T3 feeder layer in six-well plates and cultured for 14 days as previously described (Jensen et al., 2010). Feeders were removed from the keratinocyte colonies prior to fixation with 2% paraformaldehyde and staining with 1% Nile red and 1% rhodamine B blue (all Sigma). Images of stained colonies were taken on a Molecular Imager Gel Doc XR^+^ imaging system (BioRad).

### Histology and Immunohistochemistry

Tissue samples for sections were fixed overnight in 4% paraformaldehyde (Sigma) and embedded in paraffin wax. Five micrometer sections were prepared and stained with H&E.

Tail epidermal whole mounts and back skin horizontal whole mounts were prepared as described previously (Braun et al., 2003; Driskell et al., 2013). Immunohistochemistry on paraffin wax sections was performed as described elsewhere (Niemann et al., 2002). Primary antibodies used were: rat anti-BLIMP1 (1:100; eBioscience 14-5963-82), rabbit anti-CRABP2 (1:100; Proteintech 10225-1-AP), goat anti-FABP5 (1:100; R&D Systems AF1476), mouse anti-FAS (1:100; Santa Cruz Biotechnology sc-48357), rabbit anti-GFP (1:500; Life Technologies A11122), chicken anti-GFP (1:500; Abcam ab13970), goat anti-GFP (1:200; Abcam ab6673), rabbit anti-involucrin (1:800; ERLI-3, in-house), rabbit anti-K6 (1:500; Covance RBP-169P), mouse anti-K14 (1:1,000; LL002; in-house), rabbit anti-K14 (1:1,000; Covance PRB-155P), chicken anti-K14 (1:1,000; Covance SIG-3476), guinea pig-anti-K31 (1:100; Progen GP-hHa1), rabbit anti-Ki67 (1:100; Abcam ab16667), mouse anti-PCNA (1:100; Millipore CBL407), and rabbit anti-RFP (1:1,000; recognizing tdTomato; Rockland 600-401-379). Antibody staining was visualized using appropriate species-specific secondary antibodies conjugated to Alexa 488, Alexa Fluor 555, or Alex Fluor 647 (1:300; Life Technologies). LipidTOX dye (1:500; Life Technologies) was used in some cases to label lipids. Slides were mounted using ProLong Gold antifade reagent (Life Technologies) containing DAPI (Sigma) as nuclear counterstain.

All fluorescent sections were analyzed on a TCS SP5 confocal microscope (Leica) or an A1 confocal microscope (Nikon). All images of H&E-stained sections were taken on an Axiophot microscope with an AxioCam HRc camera (both Zeiss) or on an AZ100M microscope (Nikon) with a DS-Fi2 camera (both Nikon). Images were taken with constant settings optimized for each protein. Images of stained colonies were taken on a Molecular Image Gel Doc XR^+^ imaging system (BioRad).

### Quantitation and Statistics

Quantitation of IFE thickness, HF infundibulum length, and SG size was performed on images taken of H&E-stained vertical tissue sections collected from three *Blimp1* cKO and three control mice. Statistical analysis was performed using the unpaired two-tailed Student’s t test.

## Supplemental Information

Document S1. Figures S1–S4

Document S2. Article plus Supplemental Information
