# Supplementary material for: BLIMP1 Is Required for Postnatal Epidermal Homeostasis but Does Not Define a Sebaceous Gland Progenitor under Steady-State Conditions
Source: Stem Cell Reports. 2014 Sep 18;3(4):620–33. doi: 10.1016/j.stemcr.2014.08.007 (PMC4223714; doi:10.1016/j.stemcr.2014.08.007)
Supplement: Document S1. Figures S1–S4 [file mmc1.pdf]

Stem Cell Reports, Volume *3*

Supplemental Information

BLIMP1 Is Required for Postnatal Epidermal Homeostasis but Does Not Define a Sebaceous Gland Progenitor under Steady-State Conditions

Kai Kretzschmar, Denny L. Cottle, Giacomo Donati, Ming-Feng Chiang, Sven R. Quist, Harald P. Gollnick, Ken Natsuga, Kuo-I Lin, and Fiona M. Watt

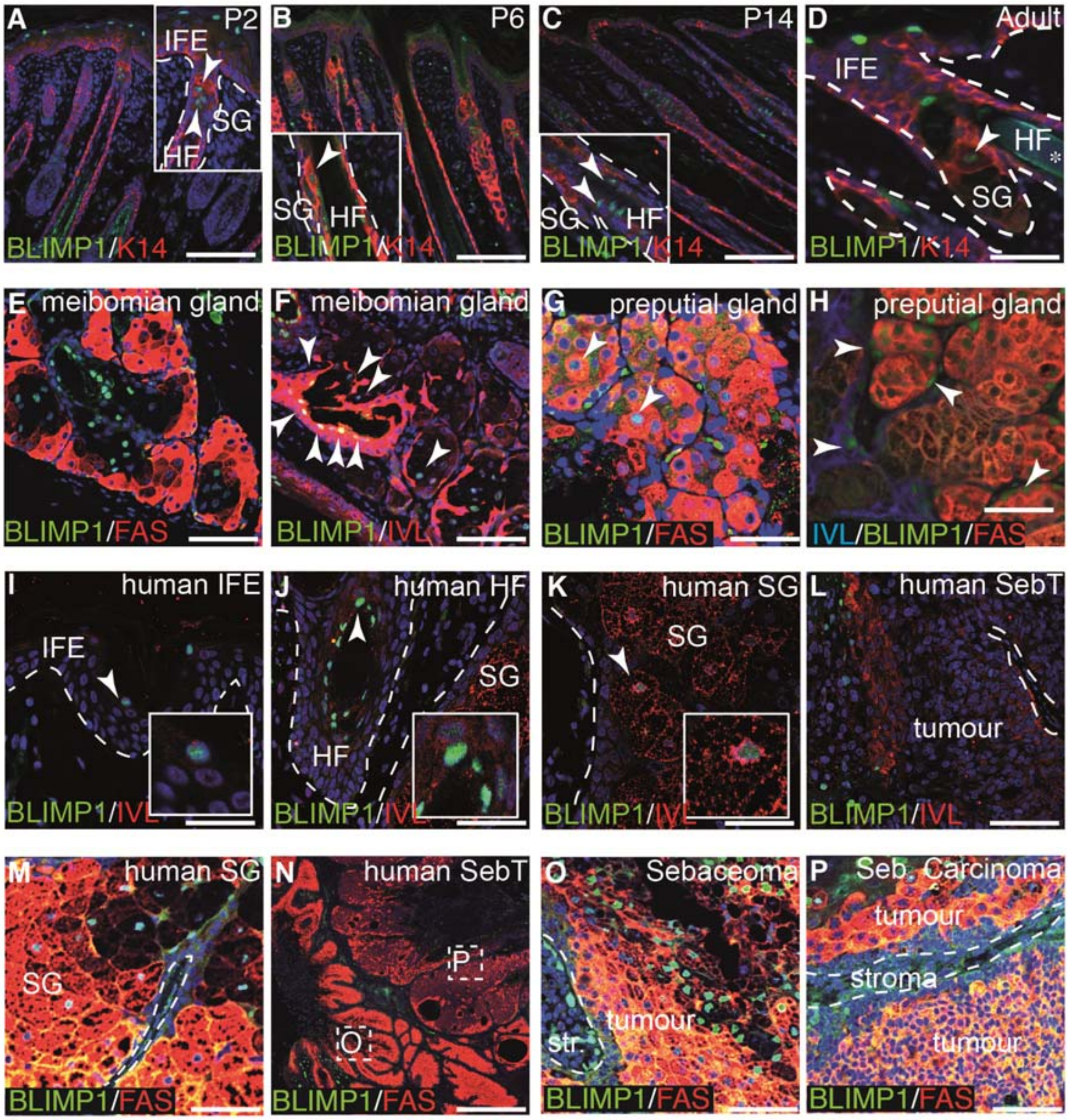

Figure S1. BLIMP1 expression in mouse and human sebocytes. Related to Figure 1. (A-D) Paraffin sections of murine wild-type back skin collected at postnatal day (P) 2, P6, P14 or P56 (adult) labelled with antibodies against BLIMP1 (green) and keratin 14 (K14; red), counterstained with DAPI (blue). (E-H) Paraffin sections of adult murine wild-type meibomian (E, F) and preputial (G, H) glands stained for BLIMP1 (green) and FAS (red in E, G, H) and IVL (red in F; blue in H) with DAPI counterstain (blue). (I-P) Paraffin sections of human skin (I-K, M) and sebaceous tumours (L-P), stained for BLIMP1 (green) and involucrin (red in I-L) and FAS (M-P) with DAPI counterstain (blue). Arrowheads: BLIMP1+ cells; dashed lines: boundary between epithelium and stroma. Boxed inserts are higher magnification views. Scale bars: 100 μm (A, B, C, E, F, I-M, O, P), 50 μm (D, G, H). 800 μm (N).

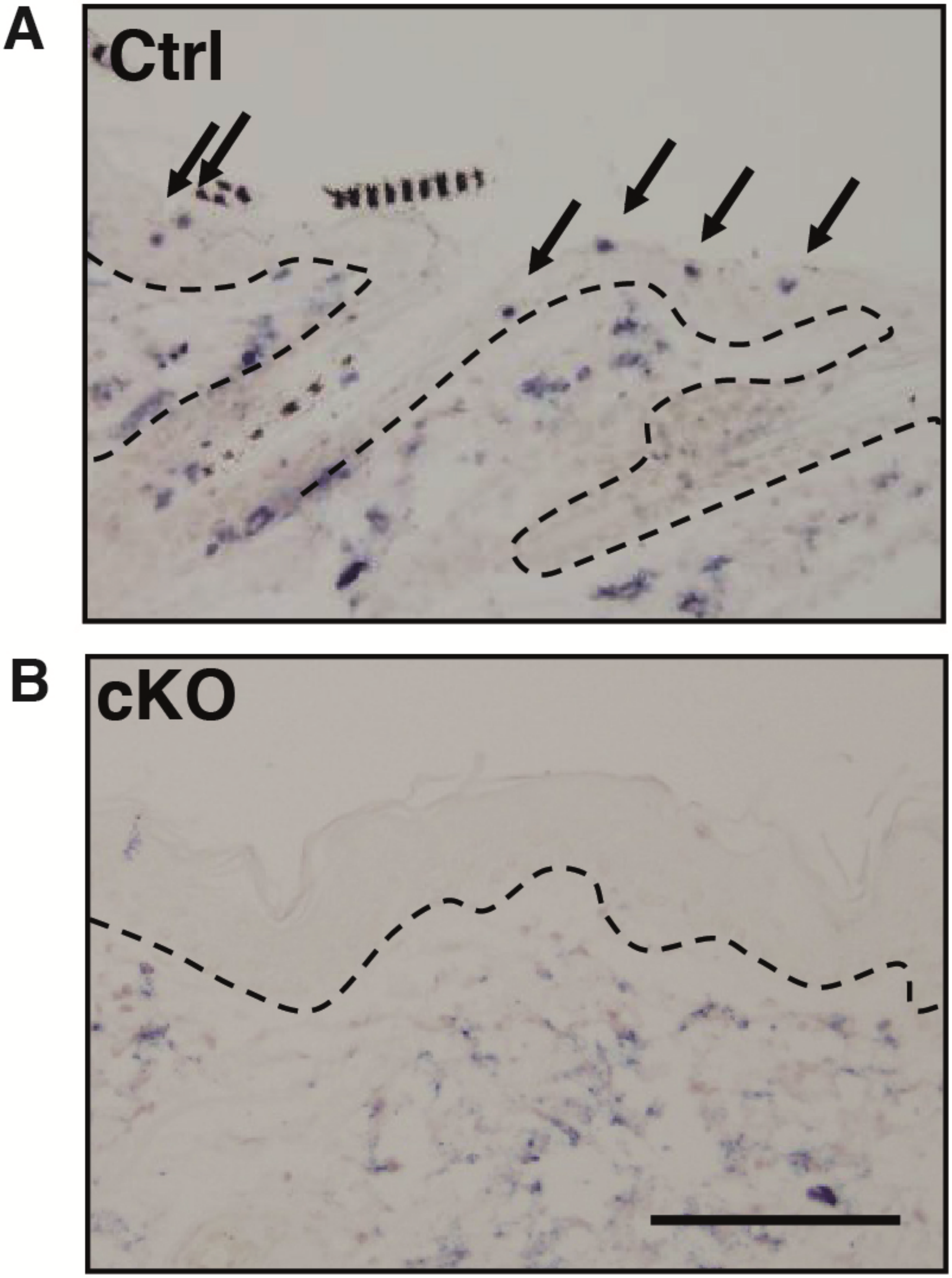

Figure S2. Confirmation of epidermal *Blimp1* deletion. Related to Figure 3. Paraffin sections of murine Blimp1 cKO and control adult back skin labelled with antibodies against BLIMP1 (blue). Scale bars: 100 μm.

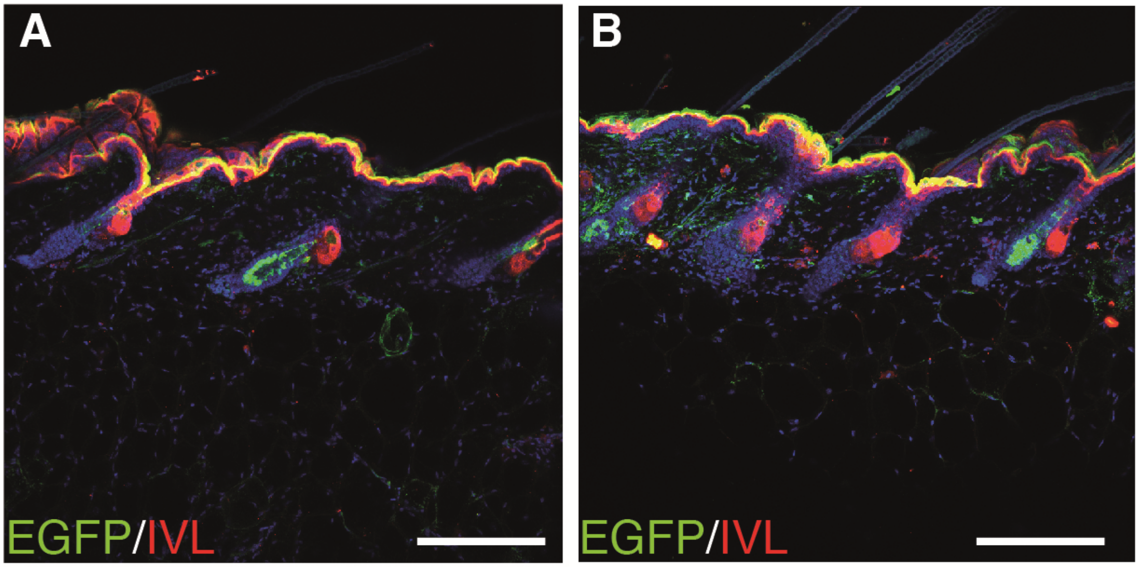

Figure S3. BLIMP1 lineage tracing in adult back skin. Related to Figure 5. Horizontal wholemounts of adult Blimp1Cre × CAGcatEGFP mice labelled with antibodies against EGFP (green) and IVL (red) and counterstained with DAPI (blue). Scale bars: 200 μm.

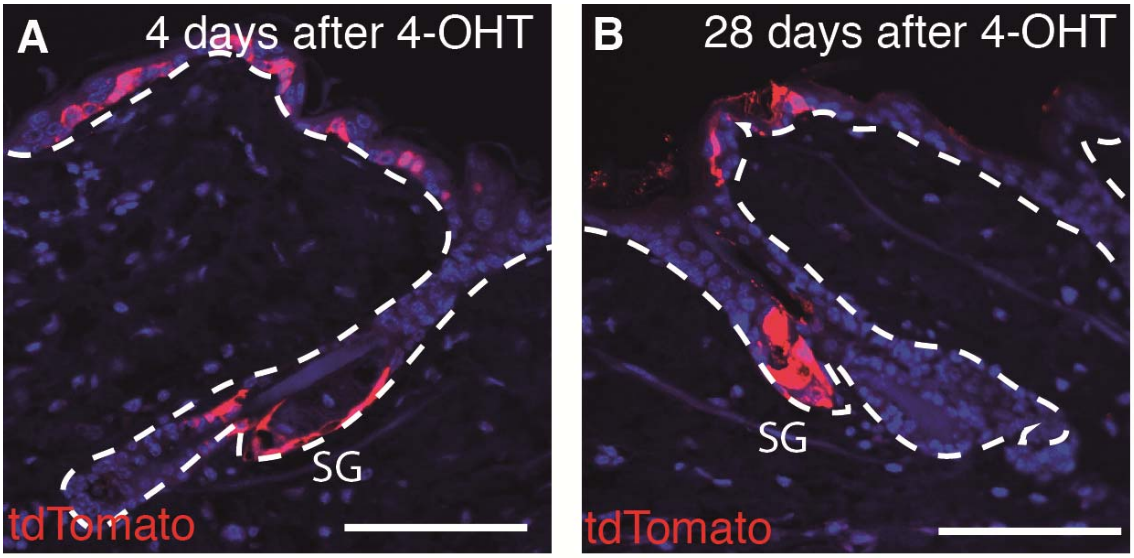

Figure S4. Lineage tracing progeny of LGR6+ stem cells in adult back skin. Related to Figure 6. Paraffin sections of *Lgr6* KI × Rosa26tdTomato skin collected four (A) and 28 (B) days after application of one dose of 4-OHT. Stained for tdTomato (red) and counterstained with DAPI (blue). Dashed lines indicate epidermal-dermal boundary. Scale bars: 100 μm.
